# Single episode of alcohol use resulting in injury: a cross-sectional study in 21 countries

**DOI:** 10.2471/BLT.17.202093

**Published:** 2018-03-20

**Authors:** Cheryl J Cherpitel, Yu Ye, Vladimir Poznyak

**Affiliations:** a Alcohol Research Group, Public Health Institute, Suite 450, 6001 Shellmound Street, Emeryville, California, CA 94608, United States of America.; b Department of Mental Health and Substance Abuse, World Health Organization, Geneva, Switzerland.

## Abstract

**Objective:**

To examine the empirical basis for including the diagnostic category of “a single episode of harmful substance use” in the 11th revision of the *International statistical classification of diseases and related health problems* (ICD-11).

**Methods:**

We used data on patients admitted to emergency departments in 21 countries with alcohol-related injuries (i.e. with drinking within the preceding six hours) who had no sign of alcohol intoxication or withdrawal, no alcohol in blood and no sign of alcohol dependence or harmful drinking as described in the ICD-10. We obtained data on alcohol-related injuries, the patient’s causal attribution of injury to drinking, the alcohol amount consumed, blood alcohol concentration and usual drinking pattern. Patients with and without alcohol dependence or harmful drinking were compared.

**Findings:**

We included a representative sample of 18 369 patients. After adjustment for unequal sampling, 18.8% reported drinking in the six hours before injury and 47.1% of these attributed their injury to drinking; 16.3% of those reporting drinking and 10.3% of those attributing their injury to drinking were not alcohol dependent or harmful drinkers. The majority of these last two groups reported never having had five or more drinks on one occasion during the last year and had a blood alcohol concentration less than 0.05%.

**Conclusion:**

Some individuals attending emergency departments had alcohol-attributable injuries due to a single episode of drinking but had no history of harmful use or dependence. These findings highlight the public health relevance of including the new diagnostic category in the ICD-11.

## Résumé

**Objectif:**

Examiner les conditions empiriques de l'inclusion de la catégorie de diagnostic «épisode isolé de consommation nocive de substances» dans la 11^e^ révision de la Classification internationale des maladies et des problèmes de santé connexes (CIM-11).

**Méthodes:**

Nous avons utilisé des données relatives à des patients admis dans des services d'urgence dans 21 pays pour des traumatismes liés à l'alcool (consommation au cours des six dernières heures) qui ne présentaient aucun signe d'intoxication à l'alcool ou de sevrage alcoolique, aucune trace d'alcool dans le sang et aucun signe de dépendance à l'alcool ou de consommation nocive, comme décrit dans la CIM-10. Nous avons recueilli des données sur les traumatismes liés à l'alcool, l'attribution causale du traumatisme à la consommation d'alcool par le patient, la quantité d'alcool consommée, la concentration d'alcool dans le sang et les habitudes de consommation. Nous avons comparé des patients qui présentaient une dépendance à l'alcool ou une consommation nocive à d'autres pour qui ce n'était pas le cas.

**Résultats:**

Nous avons inclus un échantillon représentatif de 18 369 patients. Après ajustement pour échantillonnage inégal, 18,8% des patients ont indiqué avoir consommé de l'alcool au cours des six heures ayant précédé le traumatisme, 47,1% d'entre eux attribuant leur traumatisme à la consommation d'alcool; 16,3% des patients ayant indiqué avoir consommé de l'alcool et 10,3% de ceux attribuant leur traumatisme à la consommation d'alcool n'étaient ni dépendants à l'alcool, ni des consommateurs excessifs. La majorité des membres de ces deux derniers groupes ont signalé n'avoir jamais consommé cinq verres ou plus lors d'une même occasion au cours de l'année écoulée et présentaient une concentration d'alcool dans le sang inférieure à 0,05%.

**Conclusion:**

Certains patients pris en charge par des services d'urgence avaient des traumatismes attribuables à l'alcool en raison d'un épisode isolé de consommation, mais ne présentaient pas d'antécédents de consommation nocive ou de dépendance. Ces conclusions soulignent l'intérêt pour la santé publique d'inclure une nouvelle catégorie de diagnostic dans la CIM-11.

## Resumen

**Objetivo:**

Examinar las bases empíricas para incluir la categoría diagnóstica de "episodio único de consumo de sustancia nociva" en la undécima revisión de la *Clasificación internacional de enfermedades y problemas relacionados con la salud* (CIE-11).

**Métodos:**

Utilizamos datos de pacientes admitidos en salas de emergencia en 21 países con daños asociados al alcohol (es decir, consumo de alcohol en las seis horas previas) que no presentaron signos de intoxicación por alcohol o abstinencia, sin rastros de alcohol en sangre y sin signos de dependencia del alcohol o consumo nocivo según se describe en la CIE-10. Obtuvimos datos sobre daños relacionados con el alcohol, la atribución causal del daño a la bebida realizada por el paciente, la cantidad de alcohol consumido, la concentración de alcohol en sangre y el patrón habitual de consumo. Se compararon pacientes con dependencia al alcohol y sin dependencia o consumo nocivo.

**Resultados:**

Incluimos una muestra representativa de 18 369 pacientes. Después del ajuste por muestreo desigual, el 18,8% informaron haber bebido en las seis horas previas al daño y el 47,1% atribuyó el daño a la bebida; el 16,3% de los que indicaron haber bebido y el 10,3% de los que atribuyeron el daño a la bebida no eran dependientes al alcohol o consumidores nocivos. La mayoría en estos últimos dos grupos dijo no haber bebido cinco o más tragos en una ocasión durante el último año y su concentración de alcohol en sangre fue inferior al 0,05%.

**Conclusión:**

Algunos individuos recibidos en salas de emergencia presentaron daños atribuibles al alcohol debido a un único episodio de consumo de alcohol, pero no tenían antecedentes de consumo nocivo o dependencia. Estos resultados destacan la importancia para la salud pública de la inclusión de la nueva categoría diagnóstica en la CIE-11.

## ملخص

**الغرض:**

فحص الأسس التجريبية لتضمين الفئة التشخيصية فيما يتعلق بـ "تعاطي المواد الضارة لمرة واحدة" في المراجعة الحادية عشر *للتصنيف الدولي للأمراض والمشاكل الصحية ذات الصلة * (ICD‑11).

**الطريقة:**

استخدمنا البيانات على المرضى ممن دخلوا أقسام الطوارئ في 21 دولة نتيجة لإصابات تتعلق بتناول الكحوليات (أي من تعاطى الكحوليات خلال الست ساعات السابقة) ممن لم يظهر عليهم أي علامات تدل على تسمم الكحول أو الانسحاب، ولا يوجد أثر للكحول في الدماء ولا توجد علامة تدل على إدمان الكحوليات أو المشروبات الضارة على النحو الموصوف في ICD‑10. حصلنا على البيانات بشأن الإصابات المتعلقة بالكحول، وادعاء المريض بأن سبب الإصابة يكمن في تعاطي الكحوليات، وكمية الكحوليات المستهلكة، وتركيز الكحول في الدم، ونمط الشرب المعتاد. وتم عقد مقارنة بين المرضى من مدمني الكحوليات أو ذوي العادات الضارة لتناول الكحوليات.

**النتائج:**

قمنا بتضمين عينة نموذجية تشتمل على 18369 مريضًا. بعد تعديل العينات غير المتكافئة، تم الإبلاغ عن وجود 18.8‏% من المتعاطين للكحوليات خلال الست ساعات السابقة للإصابة، وأرجع 47.1‏% من هؤلاء إصاباتهم إلى تعاطي الكحوليات؛ وأبلغ ‏16.3‏% منهم عن تعاطيهم للكحول، فيما أن ‏10.3‏% ممن أرجعوا إصابتهم إلى تعاطي الكحوليات لم يكونوا من مدمني الكحوليات ولا ممن يتناولون الكحوليات بطريقة كفيلة بالإضرار بصحتهم. وتبين أن الأغلبية من المجموعتين الأخيرتين لم يشربوا خمس مرات أو أكثر في مناسبة واحدة خلال العام السابق وبلغت نسبة تركيز الكحول في الدم أقل من 0.05‏%.

**الاستنتاج:**

تعرض بعض الأفراد ممن دخلوا أقسام الطوارئ إلى إصابات تتعلق بتناول الكحوليات نتيجة لتعاطي الكحول مرة واحدة ولم يكن لهم تاريخ سابق بإدمان الكحول أو تعاطيه. وتسلط هذه النتائج الضوء على ارتباط الصحة العامة بتضمين الفئة التشخيصية الجديدة في ICD‑11. ****

## 摘要

**目的:**

旨在调查将“有害物质使用的单次发作”诊断类别纳入第 11 版《疾病和有关健康问题的国际统计分类》(ICD-11) 的经验基础。

**方法:**

我们运用来自 21 个国家因饮酒致伤（即饮酒后 6 个小时之内）而进入急诊部的患者数据。根据第 10 版《疾病和有关健康问题的国际统计分类》(ICD-10) 的描述，这些患者无酒精中毒或者戒断迹象、血液中无酒精、无酒精依赖症或者有害饮酒的迹象。我们获取有关酒精相关伤害、患者饮酒致伤的因果归因、摄入的酒精量、血液中的酒精浓度和通常饮酒模式的数据。我们对有无酒精依赖症或者是否为有害饮酒的患者进行了对比。

**结果:**

我们纳入了 18369 份患者样本。对不等概率抽样进行整理后，18.8% 的患者称受伤之前 6 个小时内饮过酒；其中 47.1% 的患者将其受伤归因为饮酒；16.3% 的患者称饮过酒，而且将受伤归因为饮酒的患者中，10.3% 的患者没有酒精依赖症或者不是有害饮酒者。以上两组患者中，大多数患者表示过去一年从未在一种场合喝过五杯或以上的酒，而且血液中的酒精浓度低于 0.05%。

**结论:**

一些因饮酒致伤进入急诊部的患者是由于饮酒单次发作，但其并无有害饮酒或者酒精依赖症病史。这些结果突出了将新的诊断类别纳入《疾病和有关健康问题的国际统计分类》(ICD-11) 的公共卫生相关性。****

## Резюме

**Цель:**

Изучить эмпирическую основу для включения диагностической категории «единичного эпизода употребления вредных веществ» в 11-ю редакцию *Международной классификации болезней и проблем, связанных со здоровьем* (МКБ-11).

**Методы:**

Авторы использовали данные о пациентах, поступивших в отделения неотложной помощи в 21 стране с травмами, полученными после приема алкоголя (например, при потреблении алкоголя в течение предыдущих шести часов), у которых не было признаков алкогольного опьянения или синдрома абстиненции при отсутствии алкоголя в крови и признаков алкогольной зависимости или злоупотребления алкоголем, как описано в МКБ-10. Авторы получили данные о травмах, связанных с приемом алкоголя, причинно-следственных связях получения травмы и употребления алкоголя пациентом, количестве потребленного алкоголя, концентрации алкоголя в крови и обычной модели потребления алкоголя. Было проведено сравнение пациентов с алкогольной зависимостью и без нее.

**Результаты:**

Авторы включили в исследование репрезентативную выборку из 18 369 пациентов. После корректировки неоднородной выборки были получены следующие данные: 18,8% респондентов сообщили о том, что употребляли алкоголь за шесть часов до получения травмы, и 47,1% из них считали получение травмы следствием приема алкоголя; 16,3% из тех респондентов, которые сообщили о приеме алкоголя, и 10,3% из тех, кто считал получение травмы следствием приема алкоголя, не имели алкогольной зависимости и не злоупотребляли алкоголем. Большинство респондентов из этих двух последних групп сообщили, что за последний год не имели случаев единовременного употребления пяти или более порций алкоголя и концентрация алкоголя в их крови составляла менее 0,05%.

**Вывод:**

Некоторые пациенты, поступившие в отделения неотложной помощи, имели травмы, полученные после приема алкоголя, по причине единственного эпизода употребления алкоголя, но не имели никакой истории злоупотребления алкоголем или алкогольной зависимости. Эти результаты подчеркивают актуальность для общественного здравоохранения включения новой диагностической категории в МКБ-11.

## Introduction

Alcohol use is among the top 10 risk factors for ill health globally and is one of the five leading risk factors among men, such use accounts for 3.9% to 5.1% of the global disease burden.^1^^,^^2^ Injury constitutes a major part of this burden: 24.4% to 25.8% of all deaths attributable to alcohol and 30.7% to 33.2% of all alcohol-attributable disability-adjusted life years lost are due to injuries.^2^^,^^3^ One public health strategy for reducing the disease burden is to ensure that effective interventions targeting alcohol use are provided by health services,^4^ especially by emergency departments and trauma centres, because many health conditions presented at admission are associated with alcohol.^5^ However, the casual role of alcohol use in injuries is often unrecognized, particularly when there is no marked alcohol intoxication. Moreover, health professionals in busy emergency departments may have little time to assess a patient’s history of alcohol use or to diagnose alcohol use disorders.

The *International statistical classification of diseases and related health problems, 10th revision* (ICD-10) includes codes for alcohol use disorders that are commonly used for alcohol-focused interventions: acute alcohol intoxication (F10.0), harmful use of alcohol (F10.1) and alcohol dependence (F10.2).^6^ However, episodes of alcohol use that cause harm (e.g. alcohol-related injury) but cannot be described using these codes can neither be diagnosed nor classified using the ICD-10. During the development of the 11th revision of the ICD (ICD-11), a primary objective was to improve the clinical utility of the classification of mental and behavioural disorders.^7^ One proposed innovation is the inclusion of a new diagnostic category for a “single episode of harmful use” of psychoactive substances, which would help identify and document episodes of substance use that result in harm in the absence of a sustained harmful pattern of use or of substance dependence.^8^ Inclusion of this new category in the draft ICD-11 has two objectives. First, to facilitate the identification of patients in whom substance use has caused a health condition but who have no clear clinical manifestations of substance intoxication or substance dependence. Second, to encourage the provision of substance-focused interventions for these patients, such as brief interventions, in diverse health-care settings, including emergency departments.

The association between alcohol use and injury has most often been studied in patients attending emergency departments.^9^ One difficulty has been identifying those with alcohol-related injuries. Self-reported drinking in the six hours before the event that caused the injury has typically been used as indicator of alcohol-related injury and has been found to be valid when compared with objective measures such as the blood alcohol concentration.^10^ A more stringent criterion in patients who report drinking in the preceding six hours is their attribution of a causal association between drinking and the injury event.^11^ However, attribution is influenced by both the volume of alcohol consumed before the injury and by how much and how often the individual usually drinks.^12^ An individual’s usual drinking pattern is a good predictor of harmful outcomes – episodic, heavy consumption is considered the most detrimental pattern for health.^13^ An analysis of patients presenting with injuries to emergency departments across 19 countries found that, although the volume consumed predicted alcohol-related injury, both episodic, heavy drinking and frequent, heavy drinking were better predictors than other drinking patterns.^11^

The aim of this study was to examine the empirical basis for including the diagnostic category of a “single episode of harmful substance use” in the ICD-11 by examining data from a large international data set on injuries involving alcohol.^14^ We determined the proportion of patients presenting to emergency departments with alcohol-related injuries who had no sign of alcohol intoxication or withdrawal, a low or zero blood alcohol concentration and no sign of alcohol dependence or harmful drinking as described in the ICD-10. We report how alcohol-related injury and the causal attribution of injury to drinking were associated with the amount consumed before the injury, the blood alcohol concentration and the usual drinking pattern in patients with and without alcohol dependence or harmful drinking.

## Methods

Our analysis included data on a representative sample of 18 369 injured patients who attended 50 emergency departments in 21 countries (Table 1) that took part in the International Collaborative Alcohol and Injury Study, which comprised four international, collaborative research projects on alcohol and injury: the Emergency Room Collaborative Alcohol Analysis Project (ERCAAP) and three studies conducted by the World Health Organization (WHO), the Pan American Health Organization (PAHO) and the National Institute on Alcohol Abuse and Alcoholism (NIAAA) in the United States of America. All used similar methods,^20^ The studies involved probability samples of consecutive injured patients aged 18 years or older who arrived in emergency departments within six hours of an event that caused injury. Each time of day (i.e. shift) and each day of the week was equally represented.

**Table 1:** Injured patients in emergency departments who drank before injury and who attributed their injury to drinking, worldwide, 2001–2015

Region and country	Study city and reference	Year of study	Emergency departments	Injured patients	Patients who drank before injury^a^	Patients who attributed their injury to drinking
			No.	No.	%	%^b^
**Africa**						
Mozambique	Maputo	2001	1	459	16.7	29.2
South Africa	Cape Town^5^	2001	1	464^c^	45.3	51.7
United Republic of Tanzania	Moshi (unpublished data, 2018)	2013–2014	1	516	27.9	22.4
**Americas**						
Argentina	Mar del Plata^5^	2001	1	452^c^	21.1	41.8
Brazil	Sao Paulo^5^	2001	1	496	12.6	36.4
Canada	Orangeville^5^	2002	1	222^c^	6.3	33.3
Canada	Vancouver^15^	2009	2	249	22.2	23.4
Canada	Vancouver and Victoria^16^	2014	3	1191^c^	14.7	25.2
Costa Rica	San Jose^17^	2012–2013	2	1013	8.7	54.0
Dominican Republic	Santo Domingo^18^	2010	1	501	19.1	44.3
Guatemala	Guatemala City^18^	2011	1	513	20.7	79.3
Guyana	Georgetown^18^	2010	1	485	20.9	43.3
Mexico	Mexico City^5^	2002	1	456	17.0	36.4
Nicaragua	Managua^18^	2010	2	518	21.5	53.3
Panama	La Chorrera, Colon and Veraguas^18^	2010	3	490	20.5	45.2
Trinidad and Tobago	Mount Hope, San Fernando, Port-of-Spain and Scarborough^17^	2015	4	252	20.5	38.6
**Europe**						
Belarus	Minsk^5^	2001	1	457	30.0	30.9
Czechia	Prague^5^	2001	1	510	7.7	23.1
Ireland	Dublin, Galway, Letterkenny, Sligo and Waterford^11^	2003–2004	6	2088	22.9	64.6
Sweden	Malmö^5^	2001	1	497	15.1	32.3
Switzerland	Lausanne^19^	2006	1	325	25.4	39.0
**Western Pacific**						
China	Changsha^5^	2001	1	533	18.8	43.3
China	Beijing, Hangzhou, Chengdu, Hengyang and Changsha^11^	2008	5	2540	15.3	40.6
China	Taipei (unpublished data, 2018)	2009	2	1035	6.4	34.9
Republic of Korea	Bucheon and Uijeoingbu^11^	2007	2	118	37.3	61.0
Republic of Korea	Seoul, Suwon, Chuncheon and Dong-gu^11^	2008–2009	4	1989	23.6	63.0
**Total**	**NA**	**NA**	**50**	**18 369**	**18.8^d^**	**47.1^d^**

NA: not applicable.

^a^ Patients who reported drinking alcohol in the six hours before injury.

^b^ The percentage of patients who drank before injury.

^c^ In this study, patients were oversampled at some times of the day and data were weighted before inclusion in the analysis.

^d^ This figure was calculated after data were weighted to adjust for unequal probability sampling in some studies.

After giving informed consent, patients were interviewed by trained interviewers and their blood alcohol concentration was assessed using a breath analyser, which gives estimates that are highly correlated with chemical blood analyses.^21^ Across all studies, 82% of patients approached agreed to be interviewed; reasons for not completing the interview included refusal, incapacitation, being in police custody, language barriers and leaving before the end of the interview. Patients who were too severely injured to be interviewed in the emergency department were approached later in hospital after their condition had stabilized. Patients also completed a 25-minute questionnaire that included items on: (i) drinking before injury; (ii) the causal association between their drinking and injury; (iii) the usual quantity and frequency of their drinking and symptoms of alcohol dependence and harmful drinking in the last year; and (iv) demographic characteristics.

We regarded an injury as alcohol-related if the patient reported consuming alcohol in the preceding six hours, this definition has a high validity when compared with the blood alcohol concentration.^10^ Interviewers asked patients who reported drinking during this time, the number and size of the drinks consumed, individually for each beverage type (including local beverages). The total alcohol volume consumed was calculated and converted into several standard drinks, where we defined a standard drink as containing 16 mL, or 12.8 g, of pure ethanol. Interviewers also asked patients who reported drinking during the six hours whether they believed the event would still have happened had they not been drinking (i.e. causal attribution of injury to drinking).

To identify each patient’s usual drinking pattern in the previous 12 months, interviewers were asking a series of questions on how often they drank alcoholic beverages, on a range from every day or nearly every day to one to five times a year. The graduated-frequency approach was used to determine how frequently they consumed 5 to 11 drinks or 12 or more drinks on one occasion,^22^ this information was used to derive the number of occasions on which they had five or more drinks in the last year. Their usual drinking pattern was based on the frequency of alcohol consumption (i.e. less than weekly, weekly or more often) and the frequency of having five or more drinks on one occasion (i.e. never, less than weekly, weekly or more often). Although the size of each drink could not be determined using the graduated-frequency questions, we estimated the ethanol content of each drink to be 15.2 to 17.8 mL (i.e. 12 to 14 g) once the size of local beverages had been taken into account.

To assess alcohol dependence, interviewers asked patients the four questions in the Rapid Alcohol Problems Screen (hereafter referred to as RAPS4):^23^ (i) Have you had a feeling of guilt or remorse after drinking? (ii) Has a friend or a family member ever told you about things you said or did while you were drinking that you could not remember? (iii) Have you failed to do what was normally expected of you because of drinking? and (iv) Do you sometimes take a drink in the morning when you first get up? A positive response to one or more items indicates alcohol dependence. This instrument was developed and tested in emergency departments by comparison with the alcohol section of the core version of the Composite International Diagnostic Interview,^24^ which was adapted to include criteria for alcohol dependence and harmful drinking or alcohol abuse from both the ICD-10 and the *Diagnostic and Statistical Manual of Mental Disorders, fourth edition*.^25^^–^^27^ The RAPS4 was found to perform as well or better than other screening instruments for alcohol dependence in both emergency departments and in the general population.^28^^–^^33^ In addition, it was also found to have good sensitivity and specificity for identifying alcohol tolerance in 13 countries.^34^ However, as the RAPS4 did not perform as well for harmful drinking or alcohol abuse as for alcohol dependence, two questions on drinking quantity and frequency were added: (i) Have you had five or more drinks on at least one occasion in the last year? and (ii) Do you drink at least once a month? A patient who answered yes to both questions was regarded as drinking harmfully. This new instrument, called the RAPS4-QF, performed well for identifying harmful drinking or alcohol abuse in both emergency departments and in the general population in Argentina,^31^ Poland^30^ and the United States.^29^^,^^33^ The RAPS4-QF also had good sensitivity and specificity for identifying heavy drinking (i.e. five or more drinks on one occasion at least monthly) across 13 countries and was not affected by regular drinking patterns in those countries.^34^^,^^35^ We regarded a positive result on the RAPS4 or positive responses to both the quantity and frequency questions as indicating alcohol abuse or harmful drinking, respectively.

### Data analysis

We divided patients who reported drinking in the six hours before injury and those who causally attributed their injury to drinking into three groups: (i) those who had a positive result on the RAPS4 (i.e. RAPS4-positive); (ii) those who had a negative result on the RAPS4 (i.e. RAPS4-negative) but positive responses to both the quantity and frequency questions (i.e. QF-positive); and (iii) those who were RAPS4-negative and had a negative response to one or both of the quantity and frequency questions (i.e. RAPS4-QF-negative). We compared these three groups to identify differences in the amount of alcohol consumed before injury, blood alcohol concentrations, usual drinking patterns, sex and age (i.e. 18–29 years, 30–49 years or older than 49 years). In four studies, researchers oversampled patients at some times of the day for logistical reasons associated with shift patterns. Consequently, we weighted the data to adjust for unequal probability sampling (Table 1).

## Results

After adjustment for unequal sampling, it was estimated that 18.8% of the 18 369 patients reported drinking in the six hours before the event that caused injury and therefore had an alcohol-related injury. Table 1 shows, for each country, the proportion who reported drinking before injury and, of those, the proportion who causally attributed their injury to drinking. The proportion who reported drinking before injury varied widely, from 45.3% in South Africa to 6.3% in Canada. Among those who reported drinking, 47.1% attributed their injury to drinking, with a large variation across countries: from 22.4% in the United Republic of Tanzania to 79.3% in Guatemala.

Table 2 shows the number of drinks consumed before injury, the blood alcohol concentration, drinking pattern in the preceding year, sex and age for patients who reported drinking before injury and for those who attributed their injury to drinking, categorized according to whether they were RAPS4-positive, RAPS4-negative but QF-positive or RAPS4-QF-negative. Among those who reported drinking before injury, 45.6% were RAPS4-positive and 38.1% were RAPS4-negative but QF-positive. Among those who attributed their injury to drinking, 52.4% were RAPS4-positive and 37.3% were RAPS4-negative but QF-positive. Among both patients who reported drinking before injury and patients who causally attributed their injury to drinking, those who were RAPS4-positive or RAPS4-negative, but QF-positive were significantly more likely than those who were RAPS4-QF-negative to have reported a large number of drinks before injury, to have a high blood alcohol concentration and to have had five or more drinks frequently in the preceding year. Over 80% of patients who were RAPS4-positive or RAPS4-negative, but QF-positive were male compared to less than 70% of those who were RAPS4-QF-negative (Table 2).

**Table 2:** Characteristics of injured patients in emergency departments who drank before injury and who attributed their injury to drinking, by alcohol dependence^a^ and harmful drinking,^b^ worldwide, 2001–2015

Characteristic	% of patients who drank before injury^c,d^ (*n* = 3521)			% of patients who attributed their injury to drinking^d^ (*n* = 1606)		
	RAPS4-positive^a^ (*n* = 1641)	RAPS4-negative, QF-positive^b^ (*n* = 1311)	RAPS4-QF-negative^e^ (*n* = 569)	RAPS4-positive^a^ (*n* = 862)	RAPS4-negative, QF-positive^b^ (*n* = 582)	RAPS4-QF-negative^e^ (*n* = 162)
**No. drinks before injury^f,g^**						
0 to 2	9.7	17.6	42.9	7.0	13.6	39.8
3 to 5	22.7	28.6	37.5	18.4	23.9	41.5
6 to 10	30.0	32.7	13.1	31.9	36.3	13.6
> 10	37.6	21.1	6.5	42.7	26.2	5.1
**Blood alcohol concentration, %^h^**						
< 0.01	15.2	25.2	36.9	10.3	22.6	40.8
0.01 to < 0.05	25.6	23.5	24.5	23.4	24.0	18.3
0.05 to < 0.10	18.8	19.8	17.3	19.8	19.9	15.4
≥ 0.10	43.4	31.6	21.3	46.5	33.5	25.5
**Drinking pattern in last year^i,j^**						
Drank less than weekly, never had ≥ 5 drinks	3.5	0.0	37.9	3.8	0.0	40.6
Drank weekly or more often, never had ≥ 5 drinks	4.6	0.0	44.9	2.5	0.0	38.0
Drank less than weekly, had ≥ 5 drinks less than weekly	15.2	24.0	17.2	15.5	22.2	21.4
Drank weekly or more often, had ≥ 5 drinks less than weekly	13.5	26.0	0.0	9.1	20.6	0.0
Drank weekly or more often, had ≥ 5 drinks weekly or more often	63.2	50.0	0.0	69.1	57.2	0.0
**Sex**^k^						
Female	15.3	18.5	31.4	15.8	17.9	30.7
Male	84.7	81.5	68.6	84.2	82.1	69.3
**Age, years^l^**						
18 to 29	41.6	47.8	40.1	38.8	46.6	46.4
30 to 49	43.9	29.0	38.5	45.6	39.6	36.3
≥ 50	14.6	13.2	21.4	15.5	13.8	17.3

QF: two questions on drinking quantity and frequency; RAPS4: four-item rapid alcohol problems screen.

^a^ Patients were regarded as alcohol dependent if they had a positive result on the four-item rapid alcohol problems screen (i.e. were RAPS4-positive).

^b^ Harmful drinking was assessed using two questions on drinking quantity and frequency (see main text for details); patients who gave positive responses to both were regarded as drinking harmfully and were designated QF-positive. Otherwise, they were QF-negative.

^c^ Patients who reported drinking alcohol in the six hours before injury.

^d^ All percentages were calculated after data were weighted to adjust for unequal probability sampling in some studies.

^e^ Patients who were RAPS4-negative and QF-negative were designated RAPS4-QF-negative.

^f^ A standard drink was defined as containing 16 mL (i.e. 12.8 g) of pure ethanol.

^g^ Patients who were RAPS4-positive or RAPS4-negative but QF-positive were significantly more likely than those who were RAPS4-QF-negative to report a large number of drinks before injury: *P* < 0.001 (*χ*^2^ test) for both those who drank before injury and those who attributed their injury to drinking.

^h^ Patients who were RAPS4-positive or RAPS4-negative but QF-positive had significantly higher blood alcohol concentrations than those who were RAPS4-QF-negative: *P* < 0.001 (*χ*^2^ test) for both those who drank before injury and those who attributed their injury to drinking.

^i^ Drinking pattern was determined from drinking frequency and how often the patient had five or more drinks on one occasion in the last year, where the volume of a drink ranged from 15.2 to 17.8 mL (i.e. 12 to 14 g) of pure ethanol across study sites.

^j^ Patients who were RAPS4-positive or RAPS4-negative but QF-positive were significantly more likely than those who were RAPS4-QF-negative to have had five or more drinks frequently in the last year: *P* < 0.001 (*χ*^2^ test) for both those who drank before injury and those who attributed their injury to drinking.

^k^ Patients who were RAPS4-positive or RAPS4-negative but QF-positive were significantly more likely than those who were RAPS4-QF-negative to be male: *P* < 0.001 (*χ*^2^ test) for both those who drank before injury and those who attributed their injury to drinking.

^l^ Patients who were RAPS4-positive or RAPS4-negative but QF-positive were significantly more likely than those who were RAPS4-QF-negative to be young: *P* < 0.001 (*χ*^2^ test) for those who drank before injury and *P* = 0.029 those who attributed their injury to drinking.

Overall, 16.3% of patients who reported drinking before injury showed no evidence of alcohol dependence or harmful drinking. In this group, 82.8% reported never having had five or more drinks on one occasion during the last year and 42.9% reported two or fewer drinks before the injury (Table 2). In addition, 36.9% had a blood alcohol concentration less than 0.01% and 24.5% had a concentration between 0.01% and 0.05%. About two thirds were male and the majority were younger than 50 years. Furthermore, 10.3% of patients who attributed their injury to drinking showed no evidence of alcohol dependence or harmful drinking. In this group, 78.6% reported never having had five or more drinks on one occasion during the last year. In addition, 81.3% reported having had five or fewer drinks before their injury (39.8% reported two or fewer drinks) and 40.8% had a blood alcohol concentration less than 0.01% (18.3% had a concentration between 0.01 and 0.05%). Again, about two thirds were male and the majority were younger than 50 years.

## Discussion

The main diagnostic categories covering alcohol-related harm in the ICD-10 include alcohol dependence, harmful drinking, alcohol intoxication and alcohol withdrawal; no classification is available for individuals not covered by one of these four categories who may have experienced harm due to a single episode of drinking. Health professional might therefore not identify these individuals as eligible for alcohol-focused interventions.

Here we show that a substantial proportion of patients (including females) were admitted to emergency departments with alcohol-related injuries after drinking relatively small amounts and had no sign of acute alcohol intoxication. These patients also had no history of heavy episodic drinking or of any other pattern of alcohol use that would qualify as an alcohol use disorder. Hence, these patients do not fall into one of the four ICD-10 diagnostic categories but who would have met diagnostic requirements for a single episode of harmful use of alcohol as described in the draft ICD-11. This draft includes a proposed diagnostic category for a single episode of harmful use of alcohol, with the definition:

A single episode of use of alcohol that has caused damage to a person’s physical or mental health or has resulted in behaviour leading to harm to the health of others. The episode of harmful use of alcohol typically involves acute harm to health, which is not limited to symptoms of acute intoxication or withdrawal and may include substance-induced mental disorders. This diagnosis should not be made if the harm is attributed to a known pattern of alcohol use.^36^

This definition excludes three diagnostic categories already in effect: harmful pattern of use of alcohol (6B81), alcohol withdrawal (6B83) and alcohol dependence (6B82).

Our analysis did not take into account harm to others resulting from the patient’s drinking before injury, as included in the proposed definition. A previous analysis of data from emergency departments in 14 countries found that the number of patients who believed an injury due to violence could be attributed to alcohol increased by 62% if they were able to include the other person’s drinking when attributing the cause.^37^ Consequently, our data may give a conservative estimate of the proportion whose injury could have been attributed to a single episode of harmful drinking.

Our findings underscore the importance of including the new diagnostic category of a single episode of harmful drinking in the ICD-11. Its inclusion would help identify patients who could benefit from a brief intervention for harmful drinking in the emergency department and would provide support for alcohol policies aimed at reducing alcohol-related harm. At present, interventions focused on alcohol use are not routinely implemented in emergency departments despite the expectation that an alcohol-related emergency admission could provide an effective learning moment for the patient to consider reducing his drinking or stopping altogether.^38^

One strength of our study is the inclusion of data from 21 countries that used identical protocols in representative samples of emergency department patients. There are some caveats, however. Patients themselves provided information on drinking before injury, on causal attribution of the injury and on usual alcohol consumption. However, previous emergency department studies found that only a very small percentage (ranging from 0.5% to 3.3%) of patients denied drinking but had a substantial blood alcohol concentration.^39^ In addition, the proportion of patients who reported having fewer than five drinks before injury and the proportion who had a blood alcohol concentration under 0.05% were similar, indicating that the patients’ reports were valid. Another consideration is that the definition of a standard drink varied considerably both within and between countries. Heavy episodic drinking is defined by WHO as the consumption of at least 60 g of ethanol on one occasion, which is slightly lower than our definition of five of more drinks on one occasion (i.e. 60 to 70 g of ethanol).^40^ Using WHO’s definition would have resulted in a slightly higher prevalence.

In conclusion, current ICD-10 codes do not cover all alcohol-related injury, although the harmful consequence of alcohol use affects the global burden of disease. Here we show that some individuals attending emergency departments had alcohol-attributable injuries but no history of harmful use or dependence. This is just one setting in which a single episode of harmful alcohol use can be identified and recognizing such episodes can have clinical and public health implications. The findings highlight the importance of including the new diagnostic category of a single episode of the harmful use of alcohol in the ICD-11.
